# 经3种ALK抑制剂及化疗治疗的1例非小细胞肺癌病例报道

**DOI:** 10.3779/j.issn.1009-3419.2021.101.17

**Published:** 2021-05-20

**Authors:** 珏 王, 晓燕 徐, 岩岩 孙, 卉 李

**Affiliations:** 010017 呼和浩特，内蒙古自治区人民医院肿瘤内科 Department of Oncology, Inner Mongolia People's Hospital, Hohhot 010017, China

**Keywords:** *EML4-ALK*融合基因, AIK抑制剂, 肺肿瘤, *EML4-ALK* fusion gene, ALK inhibitor, Lung neoplasms

## Abstract

棘皮动物微管相关蛋白4（echinoderm microlubule-associated protein-like 4, *EML4*）和间变淋巴瘤激酶基因（anaplastic lymphoma kinase, *ALK*）可发生断裂后融合为*EML4-ALK*基因。对于*EML4-ALK*阳性非小细胞肺癌（non-small cell lung cancer, NSCLC），一代至三代的ALK抑制剂的使用已显示出显著疗效，很多患者可以从药物治疗中实现长期获益，部分患者在优化的治疗模式下可取得超过7年的长期生存。本例患者为*EML4-ALK*融合基因阳性肺腺癌，治疗结局却明显不同于其他*EML4-ALK*融合基因阳性肺癌患者，经过一代至三代ALK抑制剂靶向治疗和化学治疗疾病进展快，耐药时间短，生存期短，获益有限。患者2019年7月15日开始先后口服克唑替尼、色瑞替尼、劳拉替尼靶向治疗，行贝伐珠单抗联合培美曲塞、卡铂化疗2个疗程，于2020年9月10日患者死亡，生存期15个月。同时治疗中表现出ALK抑制剂的常见不良反应。本文分析本例患者的治疗疗效及治疗困境，为*EML4-ALK*融合基因阳性肺癌患者治疗提供探索方向。

## Abstract (en)

The echinoderm microtubule associated protein-like 4 (*EML4*) and anaplastic lymphoma kinase (*ALK*) were fractured and fused to become *EML4-ALK*. Most of these *EML4-ALK*-positive non-small cell lung cancer patients respond well to the ALK inhibitor. Many patients can benefit from drug target therapy for a long time, and some patients can achieve long-term survival of more than 7 years under the optimized treatment mode. This patient has lung adenocarcinoma positive for *EML4-ALK* fusion gene, but the treatment outcome is obviously different from that of other patients with lung cancer positive for *EML4-ALK* fusion gene. After the first to third generations of ALK inhibitor targeted therapy and chemotherapy, the disease progresses rapidly, the drug resistance time is short, the survival time is short, and the benefit is limited. The patient received targeted therapy of Crizotinib, Ceritinib and Lorlatinib successively from July 15, 2019, followed by two chemotherapy courses of Bevacizumab combined with Pemetrexed and Carboplatin. The patient died on September 10, 2020, with a survival of 15 months. At the same time, the treatment showed common adverse reactions of ALK inhibitors. This paper analyzed the therapeutic effect and treatment dilemma of this patient, and provided an exploration direction for the treatment of patients with *EML4-ALK* fusion gene positive lung cancer.

## 1 病例资料

患者女性，51岁，因“左侧胸痛伴咳嗽、咳痰、痰中带血1月”于2019年6月就诊我科，体力状态评分1分。患者吸烟5支/d，20年，入院查体阳性体征：左肺呼吸音低。完善检查，血常规、血生化未见明显异常，肺系统肿瘤标志物细胞角蛋白片段-211（cytokeratin fragment -211, Cyf-211）：3.78 ng/mL，余正常范围内。2019年6月16日胸部计算机断层扫描（computed tomography, CT）检查（图 1A）示：左肺门及左肺上叶占位（大小：76 mm×60 mm），考虑为恶性，与左肺动脉关系密切；左肺上叶癌性淋巴管炎；左肺散在结节，不除外转移；纵隔6区淋巴结肿大；左侧少许胸腔积液，右侧第2-4肋陈旧性骨折。我院气管镜检查病理活检示：双向分化、低度恶性的间叶组织来源肿瘤，细胞、血管较丰富，考虑上皮样血管内皮细胞瘤或血管肉瘤，请院际会诊。中国医学科学院肿瘤医院病理会诊并加做免疫组化后示：少许肿瘤组织，呈上皮样和短梭形态，伴坏死，形态提示低度恶性可能，免疫组化未能进一步提示分化，建议必要时重取活检。中国医学科学院肿瘤医院气管镜检查，活检后急性大出血，急诊介入止血治疗。进一步病理活检结合免疫组化示：结合免疫组化考虑肺腺癌可能性大。返回我院完善基线检查，盆腹腔增强CT、头颅增强磁共振成像（magnetic resonance imaging, MRI）、全身骨显像未见明显异常，中国医学科学院肿瘤医院活检组织基因检测提示棘皮动物微管相关蛋白4（echinoderm microlubule-associated protein-like 4, *EML4*）和间变淋巴瘤激酶基因（anaplastic lymphoma kinase, *ALK*）融合基因（*EML-ALK*）突变，初步诊断：左肺腺癌，cT4N2M0 IIIb期，*ALK*融合突变阳性。

**图 1:**
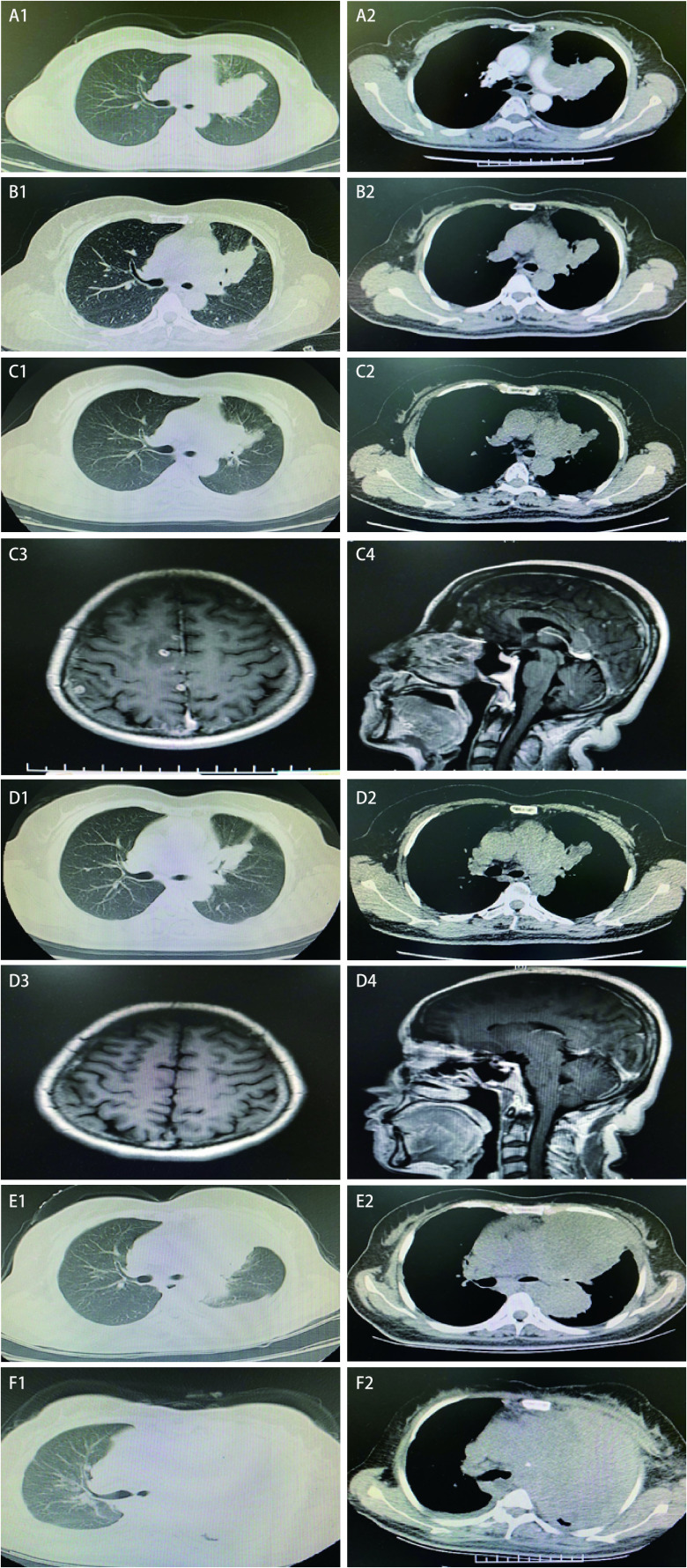
历次胸部CT及两次头颅MRI扫描图像。A：胸部CT检查（2019年6月16日）：左肺门及左肺上叶占位（大小：76 mm×60 mm），考虑恶性，与左肺动脉关系密切；左肺上叶癌性淋巴管炎；左肺散在结节，不除外转移；纵隔6区淋巴结肿大；左侧少许胸腔积液；B：胸部CT检查（2019年9月10日）：左肺门及左肺上叶肿块，伴阻塞性改变，较前肿块明显缩小（大小：49 mm×33 mm）；左肺上叶癌性淋巴管炎，较前改善；原纵隔多发增大、融合淋巴结，考虑转移，较前明显缩小；左肺下叶及叶间裂微结节，较前无显著改变；C：胸部CT（2019年11月16日）：左肺门及左肺上叶肿块，伴阻塞性改变，较前2019年9月10日进展（大小：63 mm×47 mm）；左肺上叶癌性淋巴管炎可能，较前无显著改变；纵隔多发增大淋巴结，考虑转移，6区淋巴结较前增大。头颅MRI（2019年11月18日）：颅内额叶、顶叶、枕叶及右侧脑室旁多发转移瘤；D：胸部CT（2020年1月3日）：左肺门及左肺上叶肿块，伴阻塞性改变，较2019年11月16日肿块稍减小（大小：56 mm×40 mm）；左肺上叶癌性淋巴管炎可能，较前无显著改变；纵隔多发增大淋巴结，考虑转移，较前无明显改变。头颅MRI（2020年1月5日）：颅内多发转移瘤较前明显减少、缩小；E：胸部CT（2020年7月3日）：左肺门巨大肿块伴阻塞性肺不张，较2020年5月20日明显增大（大小：87 mm×139 mm）；F：胸部CT（2020年8月27日）提示肿块增大（大小：122 mm×185 mm），纵隔多发增大淋巴结，部分较前增大，左侧胸膜结节样增厚，较前进展，双侧胸腔积液。 CT images of the chest and MRI images of the brain. A: left hilum and left upper lobe lung tumor (size: 76 mm×60 mm), considered malignant, and closely related to the left pulmonary artery; Carcinomatous lymphangitis of the upper lobe of the left lung; Sporadic nodules in the left lung; Mediastinal region 6 lymph node enlargement; A little pleural effusion on the left side (June 16, 2019); B: left hilum and left upper lobe mass, with obstructive changes, significantly smaller than the previous mass (size: 49 mm×33 mm); Carcinomatous lymphangitis of the upper lobe of the left lung was improved. Multiple enlarged and fused lymph nodes in the primary mediastinum were significantly smaller than before. Micronodules of the left lower lobe and interlobar fissure were not changed (September 10, 2019); C: tumor in left hilum and upper lobe of left lung, with obstructive changes, progressing compared with the images of September 10, 2019 (size: 63 mm×47 mm); Carcinomatous lymphangitis of the upper lobe of the left lung is no change. Multiple enlarged lymph nodes in the mediastinum were larger in zone 6 than before (November 16, 2019). Multiple metastases in the frontal lobe, parietal lobe, occipital lobe and right ventricle (November 18, 2019); D: left hilum and left upper lobe tumor slightly smaller than the images of November 16, 2019 (size: 56 mm×40 mm); Carcinomatous lymphangitis of the upper lobe of the left lung is no change. Multiple enlarged lymph nodes in the mediastinum were no significant changes (January 3, 2020). Multiple intracranial metastases were significantly smaller and disappeared compared with before (January 5, 2020); E: left hilar tumor with obstructive atelectasis, significantly larger than May 20, 2020 (size: 87 mm×139 mm) (July 3, 2020); F: an enlarged tumor, multiple enlarged lymph nodes in the mediastinum, some of which were larger than before, nodular thickening of the left pleura, more advanced than before, and bilateral pleural effusion (August 27, 2020). CT: computed tomography; MRI: magnetic resonance imaging.

第一次多学科会诊后建议患者行同步放化疗治疗，由于近期急性大出血史，患者本人抗拒放化疗治疗。依据经典临床试验结论^[1]^及临床治疗指南，2019年7月15日开始克唑替尼胶囊250 mg、每日两次口服一线治疗，同时进行患者教育，观察及定期随访观察治疗相关不良反应和疗效。患者基础肝脏功能正常范围内，服药4周后出现转氨酶陡然升高，天门冬氨酸转移酶及丙氨酸转移酶均升高，以丙氨酸转移酶升高为著，达1, 225 U/L，立即停用克唑替尼胶囊口服，予以静脉及口服应用还原型谷胱甘肽注射液、异甘草酸镁注射液及双环醇片治疗，1周后复查转氨酶明显下降，2周后异常指标恢复至正常范围内。2019年9月9日开始克唑替尼胶囊250 mg、每日一次减量口服靶向治疗，服药期间定期监测转氨酶一直在正常范围内。靶向治疗近2个月于2019年9月10日复查胸部CT（图 1B）提示左肺门及左肺上叶肿块，伴阻塞性改变，较前肿块明显缩小（大小：49 mm×33 mm）；左肺上叶癌性淋巴管炎，较前改善；原纵隔多发增大、融合淋巴结，考虑转移，较前明显缩小；左肺下叶及叶间裂微结节，较前无显著变化；左侧部分肋骨陈旧性骨折，请结合临床。疗效评价为部分缓解。我们预想患者口服克唑替尼可以较长时间获益，但在2019年11月16日再次随访复查时胸部CT示：左肺门及左肺上叶肿块，伴阻塞性改变，较前2019年9月10日进展（大小：63 mm×47 mm）；左肺上叶癌性淋巴管炎可能，较前无显著变化；纵隔多发增大淋巴结，考虑转移，6区淋巴结较前增大。更不幸的是同时复查头颅MRI提示颅内额叶、顶叶、枕叶及右侧脑室旁多发转移瘤。原发病灶及中枢神经系统均出现进展（图 1C），评价疗效为疾病进展，患者的临床分期为IVb期，此时血液下一代测序（next-generation sequencing, NGS）检测未发现阳性突变。患者体力状态评分仍为1分。

针对一代ALK抑制剂耐药的临床研究^[2]^提示，克唑替尼治疗后基因检测为野生型的耐药患者接受第二代ALK抑制剂治疗仍可达到高缓解率，客观反应率高达48%-71%，证实无*ALK*点突变的克唑替尼耐药肿瘤对第二代ALK抑制剂也有应答。依据塞瑞替尼临床研究结果^[3]^及亚裔人群的耐受性情况，2019年11月27日患者开始口服塞瑞替尼胶囊450 mg、每日一次靶向治疗，服药3 d后患者出现轻度恶心、干呕的胃肠道反应，予以口服甲氧氯普胺片及昂丹司琼片对症治疗，2周后症状明显好转。服药1个月左右，2020年1月3日复查胸部CT提示左肺门及左肺上叶肿块，伴阻塞性改变，较2019年11月16日肿块稍减小（大小：56 mm×40 mm）；左肺上叶癌性淋巴管炎可能，较前无显著变化；纵隔多发增大淋巴结，考虑转移，较前无明显改变。头颅MRI检查提示颅内多发转移瘤较前明显减少、缩小。评价疗效为疾病稳定（图 1D）。考虑患者克唑替尼治疗后疾病很快进展的临床特点，此时我们进行了第二次多学科会诊，建议在原发病灶及脑转移病灶控制稳定的情况下，加做针对上述病灶的局部放射治疗，临近春节，患者本人拒绝。新型冠状病毒抗疫期间，2020年3月26日随访复查胸部CT提示原发病灶缓慢进展（大小：60 mm×48 mm），患者仍拒绝局部治疗。

2020年4月底患者逐渐出现活动后气短、咳嗽、咳痰，2020年5月20日复查胸部CT提示左肺门及左肺上叶肿块及阻塞性肺不张，较前2020年3月26日明显增大（大小：77 mm×63 mm），头颅MRI提示脑转移病灶较前无明显变化。肿瘤标志物检查示癌抗原125（cancer antigen 125, CA125）明显升高至90.68 U/mL，评价疗效为疾病进展，体力状态评分为2分。再次血液NGS检测发现*EML4-ALK*融合V3变异体突变。劳拉替尼国内尚未上市，家属积极联系购买劳拉替尼片。考虑患者疾病进展快，症状明显，说服患者2020年6月4日开始贝伐珠单抗联合培美曲塞及卡铂化疗，治疗前血液学检查提示患者白细胞高达57.31×10^9^/L，相关科室会诊，完善感染相关指标及骨髓穿刺活检检查除外感染，考虑类白血病反应。化疗1个周期后患者白细胞下降至31.25×10^9^/L，气短症状有所好转。然而化疗2个周期后休息期间，患者气短再次加重，同时出现乏力、纳差，此时体力状态评分为3分。2020年7月3日复查胸部CT（图 1E）提示左肺门巨大肿块伴阻塞性肺不张，较2020年5月20日明显增大（大小：87 mm×139 mm），疗效评价为疾病进展。此时患者家属购买到了劳拉替尼片，2020年7月13日开始口服劳拉替尼片100 mg、每日一次靶向治疗。

口服劳拉替尼第2天患者即出现以颜面为主的轻度水肿，予以口服螺内酯片及氢氯噻嗪片间断利尿治疗，水肿在1周后好转，自觉可耐受，此次体力状态评分好转为2分。服药3周后血常规检查提示白细胞下降至11.07×10^9^/L，而且患者一般状况明显好转，主诉无明显不适，外出旅游度假，体力状态评分为1分。然而好景不长，2020年8月底患者再次出现气短、憋气明显，伴周身水肿，以左侧颜面、肢体为著，2020年8月27日胸部CT（图 1F）示肿块再次增大，左肺上叶已被肿瘤组织完全侵犯，纵隔受侵明显，合并上腔静脉综合征，积极对症支持治疗无明显疗效，于2020年9月10日临床死亡。

该患者确诊肺癌后接受了一代ALK抑制剂、二代ALK抑制剂、抗血管生成治疗联合化疗、三代ALK抑制剂治疗，在药物治疗上可谓是非常充分，然而患者的生存期只有15个月，与我们期待的“钻石突变”使用ALK抑制剂可以带来长期生存相去甚远。一代ALK抑制剂治疗只有5个月的无进展生存时间，二代ALK抑制剂有7个月的无进展生存时间，化疗仅为1个月的无进展生存时间，即使是昂贵的三代ALK抑制剂也仅有2个月的无进展生存时间。治疗期间患者出现了ALK抑制剂的一些常见不良反应，经过我们的早期识别和积极处理得到有效控制，但是值得我们深思的是患者的治疗效果难如人意，是我们需要关注的问题，也是我们临床医生在实践中的难点和挑战。

## 2 讨论

肿瘤诊疗已经迈入精准治疗时代，靶向药物的问世使具有基因突变的晚期非小细胞肺癌（non-small cell lung cancer, NSCLC）患者生存期得以不断延长，靶向治疗在晚期肺癌中的作用已得到充分肯定。目前权威指南均将分子靶向治疗列为具有基因突变的Ⅳ期肺腺癌患者的首选治疗策略。重视病理活检、动态基因检测、多学科合作模式共同助力晚期NSCLC患者的治疗。*EML4-ALK*融合基因是肺腺癌最具特点的致癌基因，我国晚期NSCLC患者*ALK*阳性的发生率为6.6%-7%^[4]^，对于无法手术的晚期*ALK*阳性NSCLC患者，依托活检和动态基因检测通过一代至三代的靶向ALK-TKI药物的合理序贯使用，长期生存已非罕见，但也有部分患者的治疗反应很差甚至无反应^[5]^。

本例患者初诊时通过活检确诊，基因检测提示具有*ALK*突变阳性，后续治疗中继续动态检测指导治疗，体现出肺癌精准治疗的精髓。但患者就诊时疾病分期偏晚，治疗过程中疾病控制时间有限，疾病变化快，通过一代至三代ALK抑制剂及化疗联合抗血管生成靶向治疗获益十分有限。Profile 1014是首个克唑替尼对比培美曲塞含铂化疗一线治疗*ALK*重排NSCLC的随机对照Ⅲ期临床研究。与化疗相比，克唑替尼治疗后疾病缓解率高（74% *vs* 45%, *P* < 0.001），中位无进展生存时间延长（10.9个月*vs* 7.0个月，HR=0.45，*P* < 0.001），疾病的复发风险下降，中位总生存期还没有达到，但是已经看到延长的趋势（NR *vs* 47.5个月，HR=0.76）^[6, 7]^。本例患者的无进展生存时间仅为5个月。多项临床研究都显示出克唑替尼序贯二代ALK-TKI治疗的中位生存期超过了4年。Gainor等^[8]^报道了克唑替尼序贯塞瑞替尼中位生存期达到49.5个月。Profile 1014研究报道克唑替尼序贯ALK-TKI治疗总生存期超过了5年^[9]^。本例患者通过一、二代ALK抑制剂序贯治疗疾病控制时间仅为12个月。劳拉替尼是第三代ALK抑制剂，是一种新型、可逆、强效小分子ATP小分子ALK和ROS1的抑制剂，对ALK已知的耐药突变均具有很强的抑制作用，对既往接受过ALK抑制剂治疗和不论是否接受化疗的患者也有较高的疾病控制率^[10]^，而该患者只有2个月的控制时间。

表达EML4-ALK的NSCLC患者表现出了对靶向药物敏感性及持续作用时间不同的情况。随着对*EML4-ALK*融合基因研究的深入，目前已经发现了超过17种*EML4-ALK*融合异形^[11]^，其中最常见的是1、2和3a/b，占总融合异形的90%^[12]^。基于对EML4-ALK分子形态的研究，学者把研究焦点转移到*EML4-ALK*异形与反应应答的相关性上。集中探讨特定融合异形是否会影响肺癌的播散和疾病的预后以及不同异形是否会影响ALK靶向治疗的疗效^[13]^。研究显示V3a/b变异体相较于V1、V2变异体对酪氨酸激酶抑制剂敏感性降低^[14]^，*EML4-ALK* V3a/b、V5a变异体患者较V1、V2变异体患者的无进展生存期明显缩短^[15]^。该患者在后续动态基因检测中检出*EML4-ALK* V3变异体，可能与患者预后较差有关。目前关于*EML4-ALK*变异体研究的瓶颈也十分突出，首先由于*ALK*阳性病例较少，即便是多中心大型临床研究所获得的数据也仅能初步明确异形的表达和后续反应，并不能记录长期应答。希望将来进一步研究不同*EML4-ALK*变异体的差异，来回答临床中不同患者表现出的治疗差异的原因，有助于发现新的针对*ALK*突变靶向治疗的NSCLC治疗策略，改善此类患者的治疗预后和生存期。
